# Is genomic screening necessary for fetuses who suffer moderate to severe tricuspid regurgitation? A case report

**DOI:** 10.1097/MD.0000000000017771

**Published:** 2019-11-27

**Authors:** Lei Liu, Xiaoqing Shi, Peng Yue, Xiaolan Zheng, Yimin Hua, Kaiyu Zhou, Yifei Li

**Affiliations:** a Department of Pediatrics, West China Second University Hospital, Sichuan University; b Ministry of Education Key Laboratory of Women and Children's Diseases and Birth Defects, West China Second University Hospital, Sichuan University, Chengdu, Sichuan, China.

**Keywords:** dilated cardiomyopathy, DSP, fetal tricuspid regurgitation, genomic sequence, TNNT2

## Abstract

**Rationale:**

Tricuspid regurgitation (TR) is a frequent finding during echocardiography screening in fetal or neonatal life, which reveals a weak association between TR and cardiac malformation. Except for structural abnormalities, dilated cardiomyopathy (DCM) ranks as the top reason for early child morbidity and mortality among all kinds of cardiomyopathy. In the early fetal stage, cardiac abnormalities detected by early fetal genetic testing followed by abnormalities on ultrasound would provide more valuable information for parents and physicians to make a better therapeutic schedule.

**Patient concerns:**

A case of severe TR was found via the fetal ultrasound screening. After birth, this child suffered severe heart dysfunction, and echocardiography confirmed a DCM phenotype within a very short time.

**Diagnosis and intervention:**

A 40-year-old female received routine fetal echocardiographic screening, which demonstrated that the fetus presented severe TR. Six months after birth, the baby experienced severe heart failure, as the EF dropped to 22% with an extremely large LV chamber. The genomic sequence had been determined, and 3 pathogenic gene mutations located in 2 genes, cardiac troponin T (TNNT2) c.548G>A, desmoplakin (DSP) c.3146C>T, and DSP c.5213G>A, were identified. Finally, the patient was diagnosed with DCM. This child received digoxin, hydrochlorothiazide, spironolactone diuresis, captopril, and L-carnitine, and the symptoms of heart failure had been controlled as the patient waited for heart transplantation.

**Outcomes:**

During the follow-up, the patient still suffered from poor heart function and an enlarged left ventricle. Concomitantly, the parents placed her on a waiting list for heart transplantation.

**Lessons:**

Fetal TR is a common phenomenon, and many studies have indicated that isolated TR is not an appropriate predictor of chromosomal abnormalities or congenital heart defects. However, according to this case, it is urgent to recommend that the mother should take advantage of free fetal DNA analysis in a maternal blood sample to obtain further molecular evidence once fetal echocardiography reveals moderate to severe TR with any maternal high-risk factors for birth defects.

## 1 Introduction

Tricuspid regurgitation (TR) is a frequent finding during echocardiography screening in fetal or neonatal life and is observed in approximately 7% of the population. Several researchers demonstrated the association between TR and cardiac malformation.^[1,2]^ Especially for some cases of moderate or severe TR, TR was proved to be associated with defects, including Ebstein's malformation, tricuspid valve dysplasia, pulmonary atresia, and atrioventricular canal malformation.^[3]^ However, most of the defects were structural abnormalities, and among the cases of severe cardiomyopathy, the defects may have manifested a normal cardiac structure during fetal life or even early life postnatally. Among all kinds of cardiomyopathy, dilated cardiomyopathy (DCM) presents the highest prevalence and poorest outcomes for children. The earlier DCM affects cardiac function, the worse the prognosis is. By taking advantage of genomic sequencing, we are obtaining a greater understanding of the molecular basis of DCM. More than 25% of patients with DCM carry a genetic mutation resulting in sudden cardiac death and the need for heart transplantation.^[4]^ Some reports revealed that fetal TR might be associated with chromosomal defects, and these reports provide clues for detecting genetic mutations among particular fetuses with TR. Therefore, cardiac abnormalities detected via early fetal genetic testing followed by abnormalities on ultrasound would provide more valuable information for parents and physicians to make a better therapeutic schedule. Here, we report a case of severe TR observed via fetal ultrasound screening, and this case progressed to DCM in a very limited time after birth. In addition, mutations of 2 dominant genes for maintaining normal cardiomyocyte hemostasis have been confirmed. This case led to review the diagnostic procedure for moderate to severe TR in fetal life in order to assess whether genetic or chromosomal analysis was necessary for such patients.

## 2 Case presentation

A 40-year-old female underwent routine fetal echocardiographic screening at the 22nd week of gestation. The pregnancy was normal without any maternal complications, such as hypertension, diabetes, eclampsia, or infections during gestation. In addition, screening for Down syndrome revealed a low risk that did not indicate a need to conduct amniocentesis for a chromosomal abnormality test. However, the fetal ultrasound demonstrated severe TR, based on Vmax = 3.8 m/s, with estimated diameters of the cardiac chambers of LV = 11 × 19 mm, RV = 19 × 24 mm, LA = 12 × 10 mm, and RA = 13 × 16 mm. Subsequently, the female was referred to a pediatric cardiologist for advice. Because the age of the female was 40, which is a high-risk factor for birth defects, and since TR is quite commonly observed in fetal ultrasound screening, the cardiologist suggested genomic sequencing analysis of free fetal DNA in maternal blood. However, the parents refused this analysis.

**Figure 1:**
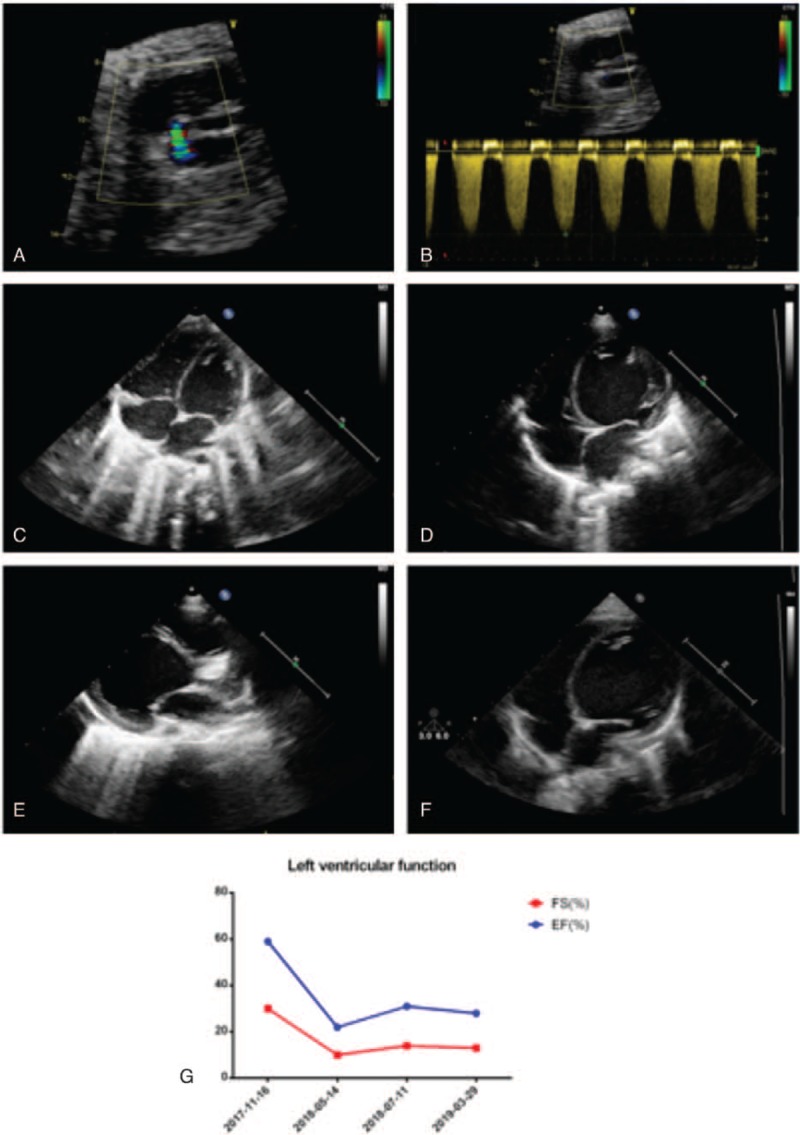
Echocardiography and the left ventricular function detection at each stage: (A-B) August 25, 2017, the fetal ultrasound showed severe tricuspid valve regurgitation, Vmax = 3.8m/s. 2D: LV = 11x19 mm, RV = 19 × 24 mm, LA = 12 × 10 mm, RA = 13 × 16 mm, AO = 5 mm, PA = 9 mm. Doppler: MV:E = 0.5 m/s, TV:E = 0.6 m/s, AV = 0.7 m/s, PV = 1.0 m/s; (C) November 16, 2017, the ultrasound showed that the size of each chamber was basically normal; (D-E) July 11, 2018, the ultrasound showed that the double ventricle was enlarged, with obvious left ventricle, slightly larger left atrium, and basically normal size of right atrium. (LV = 39 mm, RV = 15 mm, LA = 21 mm, RA = 26 mm); (F) March 29, 2019, the ultrasound showed that the left ventricle was significantly enlarged, and the size of the remaining ventricle was basically normal (LV = 40 mm RV = 8 mm LA = 18 mm RA = 28 mm); (G) the left ventricular function measurement followed the timeline.

The baby was born safely in our institute, and the first echocardiography examination postnatally demonstrated good heart function and normal structure as well as normal sizes of the 4 chambers. However, 6 months later, the baby was brought to our department due to excessive sweating and decreased milk consumption. The physical examination showed that the body temperature of the girl was 36.8°C, her heart rate was 150 beats per minute, and her respiration rate was 42 beats per minute. The blood pressure of the four limbs was as follows: 92/44 mmHg at the right upper limb, 96/58 mmHg at the left upper limb, 101/60 mmHg at the right lower limb, and 103/54 mmHg at the left lower limb. In addition, she presented a critically ill face, her lips and complexion were pale, excessive perspiration, and coarse breath were noted, but there were no rales. On auscultation, her heart boundaries were enlarged, the apex was shifted, and grade I systolic murmur was heard in the 2nd intercostal space on the left-sternal border. Routine blood examination, liver and kidney function tests, electrolytes, routine examination of urine and stool, blood gas analysis, blood ammonia analysis, pyruvate analysis, thyroid function analysis and metabolic disease detection showed no obvious abnormalities. The chest X-ray showed signs of pulmonary infiltration, and the heart size was significantly increased. Electrocardiogram (ECG) showed sinus tachycardia, axis deviation, and ST-T wave changes (II, III, AVF, decrease in current at the V5 lead not less than 0.05 mV, short T wave). Moreover, the echocardiography presented a dramatic phenotype of severe TR, an extremely enlarged left ventricle, and reduced density of the interventricular septum and left ventricular posterior wall. The LVEF dropped to 22%, and FS decreased to 10%. All the echocardiographic parameters are listed in Table 1. We excluded the possibility of myocarditis based on a series of negative blood test results and history of collections. After administration of digoxin, hydrochlorothiazide, spironolactone diuresis, captopril, and L-carnitine, the symptoms of heart failure were controlled. The genomic sequence was determined, and 3 pathogenic gene mutations located in 2 genes, cardiac troponin T (*TNNT2)* c.548G>A:p.Arg183Gln, desmoplakin (*DSP)* c.3146C>T: p.Ser1049 Leu, and *DSP* c.5213G>A: p.Arg1738Gln, were identified, all of which are very important for cardiomyocyte development and maturation postnatally (Table 2). This result could explain why this kind of DCM progressed so rapidly (Fig. 1).

**Table 1:**
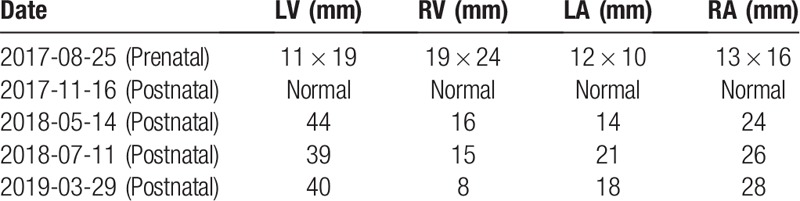
Cardiac 4 chambers diameters following timeline.

**Table 2:**
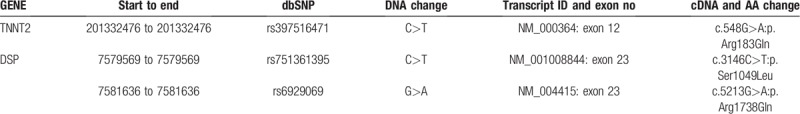
Genetic mutation information.

During the follow-up, the patient still suffered from poor heart function and an enlarged left ventricle. The latest echocardiography report (1 year and 6 months old) showed the 4 chamber sizes to be LV = 40 mm, RV = 8 mm, LA = 18 mm, and RA = 28 mm and low cardiac pump function of EF = 28% and FS = 13%. Concomitantly, the parents placed the patient on the waiting list for heart transplantation. The patient provided informed consent for publication of the case.

## 3 Discussion

Cardiomyopathy is a major class of cardiovascular diseases resulting in high morbidity and mortality.^[5]^ DCM is the most common type of cardiomyopathy. In recent years, researchers confirmed that approximately 60% of all cases of DCM are due to heredity.^[6]^ In addition, myocarditis, exposure to alcohol, drugs and toxins, and metabolic and endocrine disorders can also lead to DCM. The most common symptoms of DCM are associated with congestive heart failure but may also include circulatory failure, arrhythmias, and thromboembolic events.^[7]^ The treatment of DCM is difficult, and the lack of knowledge on the molecular or genetic etiology and pathophysiology of this disease causes physicians to be less effective in managing DCM.^[8]^

At present, several genes have been implicated in the pathogenic generation of DCM, including titin (*TTN*), beta myosin heavy chain (*MYH7*), *TNNT2*, tropomyosin (*TPM1*), myosin binding protein C3 (*MYBPC3*), filamin C (*FLNC*), *DSP*, and lamin A/C (*LMNA*).^[9]^ These genes mainly affect sarcomere proteins, cytoskeletal proteins, and ion channels. In this case, the mutant genes were *TNNT2* and *DSP*. TNNT2 is the gene encoding both fetal and adult cardiac troponin T, which has a major role in the transition from the fetal gene program to the adult gene program. TNNT2 mutation would impair the normal maturation process of cardiomyocytes, leading to rapid development of heart dysfunction postnatally. DSP is a kind of protein that exists in the desmosomes, with one end of the protein extending beyond desmin and filamin and the other end connecting to plakophilin and plakoglobin.^[9]^ Desmosomes are tightly linked cells found mainly in tissues under mechanical stress, such as the skin and heart.^[10]^ Therefore, the mutation is certain to cause heart damage. Given that, 3 mutations in 2 key genes in cardiomyocyte regulation led to a severe condition in this case.

According to this finding, we reviewed our strategy for this case. Fetal TR is a common phenomenon, and many studies have indicated that isolated tricuspid regurgitation is not a good predictor of chromosomal abnormalities and congenital heart defects,^[3,11]^ suggesting that mild TR is the result of physiological changes during pregnancy.^[12]^ In this case, the fetus presented no cardiac structural abnormalities other than severe TR. However, the mother was a 40-year-old female, which is a high-risk factor for delivery with birth defects, and she refused fetal genomic analysis. Consequently, it is urgent to recommend that the mother take advantage of analysis of free fetal DNA in the maternal blood sample to obtain further molecular evidence once fetal echocardiography reveals moderate to severe TR with any maternal high-risk factors for birth defects.

## Author contributions

**Conceptualization:** Xiaoqing Shi, Peng Yue, Yimin Hua, Yifei Li.

**Data curation:** Lei Liu, Xiaoqing Shi, Peng Yue, Yifei Li.

**Investigation:** Lei Liu, Xiaolan Zheng, Yifei Li.

**Methodology:** Lei Liu, Xiaolan Zheng, Yimin Hua, Yifei Li.

**Project administration:** Lei Liu, Xiaoqing Shi.

**Resources:** Peng Yue.

**Supervision:** Xiaoqing Shi, Yimin Hua, Kaiyu Zhou, Yifei Li.

**Validation:** Lei Liu, Xiaoqing Shi, Yifei Li.

**Writing–original draft:** Lei Liu, Peng Yue, Yifei Li.

**Writing–review & editing:** Xiaoqing Shi, Kaiyu Zhou, Yifei Li.
